# Repair of liver mediated by adult mouse liver neuro-glia antigen 2-positive progenitor cell transplantation in a mouse model of cirrhosis

**DOI:** 10.1038/srep21783

**Published:** 2016-02-24

**Authors:** Hongyu Zhang, Christopher T. Siegel, Ling Shuai, Jiejuan Lai, Linli Zeng, Yujun Zhang, Xiangdong Lai, Ping Bie, Lianhua Bai

**Affiliations:** 1 Hepatobiliary Institute, Southwestern Hospital, No. 30 Gaotanyan, ShapingBa Distract, Chongqing 400038, China; 2 Department of Surgery, Division of Hepatobiliary and Abdominal Organ Transplantation, Case Western Reserve University Hospital, Cleveland OH 44106, USA

## Abstract

NG2-expressing cells are a population of periportal vascular stem/progenitors (MLpvNG2^+^ cells) that were isolated from healthy adult mouse liver by using a “Percoll-Plate-Wait” procedure. We demonstrated that isolated cells are able to restore liver function after transplantation into a cirrhotic liver, and co-localized with the pericyte marker (immunohistochemistry: PDGFR-β) and CK19. Cells were positive for: stem cell (Sca-1, CD133, Dlk) and liver stem cell markers (EpCAM, CD14, CD24, CD49f); and negative for: hematopoietic (CD34, CD45) and endothelial markers (CD31, vWf, von Willebrand factor). Cells were transplanted (1 × 10^6^ cells) in mice with diethylnitrosamine-induced cirrhosis at week 6. Cells showed increased hepatic associated gene expression of alpha-fetoprotein (AFP), Albumin (Alb), Glucose-6-phosphatase (G6Pc), SRY (sex determining region Y)-box 9 (Sox9), hepatic nuclear factors (HNF1a, HNF1β, HNF3β, HNF4α, HNF6, Epithelial cell adhesion molecule (EpCAM), Leucine-rich repeated-containing G-protein coupled receptor 5-positive (Lgr5) and Tyrosine aminotransferase (TAT). Cells showed decreased fibrogenesis, hepatic stellate cell infiltration, Kupffer cells and inflammatory cytokines. Liver function markers improved. In a cirrhotic liver environment, cells could differentiate into hepatic lineages. In addition, grafted MLpvNG2^+^ cells could mobilize endogenous stem/progenitors to participate in liver repair. These results suggest that MLpvNG2^+^ cells may be novel adult liver progenitors that participate in liver regeneration.

Liver cirrhosis is an end-stage liver disease characterized by liver fibrosis and regenerative nodules with liver dysfunction^1^. Likely risk factors are alcohol abuse, hepatitis B virus, hepatitis C virus, hepatocellular carcinoma, inflammatory bowel disease, and smoking^2^.

For now, the treatment approaches aim at treating the underlying cause, counseling patients to stop alcohol and smoking, administering treatment for hepatitis B and C infections and at managing pain and complications. However, the only therapeutic option available at present for end-stage liver diseases and hepatic failure is orthotopic liver transplantation^3^. This approach is limited by the shortage of donor organs. Therefore, alternative treatment options are urgently needed.

Cell therapies are increasingly recognized as an important approach to facilitate functional recovery^4^,^5^,^6^. However the most effective therapeutic progenitor cell populations, such as liver stem cells, hepatic oval cells (HOC)^7^ and mesenchymal stem cells (MSCs)^8^,^9^ used to treat diseased livers remain controversial. Because of the low frequency of stem cells in adult liver^10^ and the difficulty in isolating these cells, the selective isolation of a relatively pure population of stem/progenitors from adult liver and assessment of their therapeutic potential is challenging. One hypothesis that has gained considerable attention is that neuro-glia antigen 2 (NG2)-expressing cells are found in all tissues and are closely associated with tissue vasculature^11^,^12^ and thus behave as stem/progenitors cells^13^.

The NG2 protein was originally detected by antibodies directed against surface proteins in a rat cell line with glial and neuronal properties^14^ where they are thought to play a role in regulating tissue homeostasis^15^,^16^,^17^,^18^,^19^ and the blood-brain barrier^20^,^21^. Given that NG2 is expressed by cells with stem cell-like properties, they could exhibit stem cell activities and promote functional recovery in a liver cirrhosis model^22^,^23^,^24^. An *in vivo* analysis has shown that NG2^+^ cells are closely associated with injured axons, where they could promote cell growth and increase axonal stability after spinal cord injury^25^. Recent studies have identified potential roles for the NG2-expressing cells in human liver possessing robust migratory activities and differentiation potentials^15^. It was also reported that absence of NG2 would cause obesity or fatty liver^26^. Interestingly, the evidence of neuronal stabilizing agents such as carbamazepine, an anticonvulsant drug shown to promote liver regeneration^27^, suggests that NG2^+^ cells could have a potential to promote organ regeneration.

Therefore, the aim of this study was to transplant the isolated stem/progenitors from adult mouse liver periportal vascular region by a “Percoll-Plate-Wait” procedure, into cirrhotic liver and evaluate the repair capacities of the cells in mice with liver cirrhosis.

## Results

### Characterization of MLpvNG2^+^ cells

**Figure 1:**
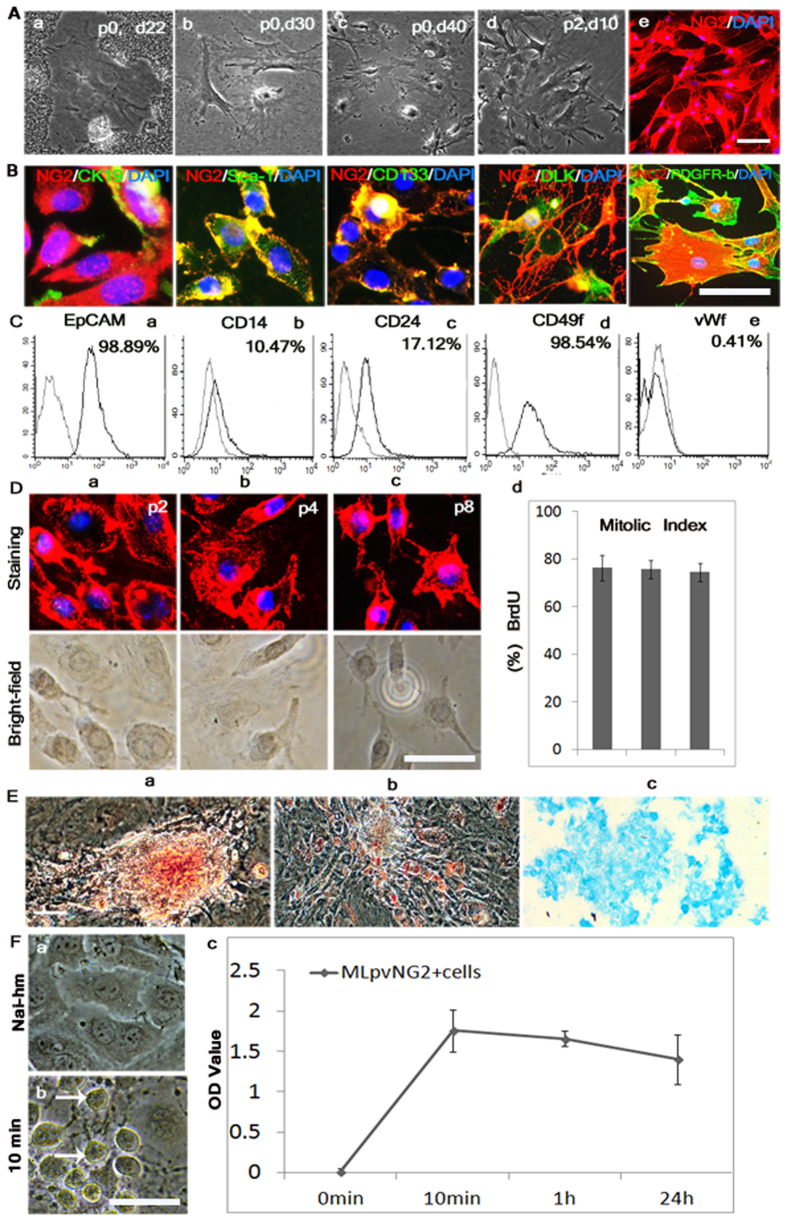
Characterization of mouse liver portal perivascular region NG2^+^ (MLpvNG2^+^) cells. (**A**a–d) cultured MLpvNG2^+^ cells at different times (P = passage, d = day). (**A**e): P2 cells were incubated with antibodies against NG2 (Red). **(B)** P2 cells were incubated with antibodies against CK19, Sca-1, CD133 DLK, and PDGFR-β (Green). (**C**a–e) EpCAM (a), CD14 (b), CD24 (c), CD49f (d) and vWf (e) were quantified by flow cytometry. (**D**a–c) Cell cultures from P2 (a), P4 (**b**) and P8 (c) stained with antibodies against NG2 (Red) corresponding with bright-field; DAPI (blue) staining was used to visualize nuclei. (**D**d) P2, P4 and P8 of MLpvNG2^+^ cells proliferation using BrdU incorporation. Proliferation was assessed by quantifying the number of BrdU-positive cells as a proportion of the total number of NG2^+^ cells (**E**a–c) Differentiation of the MLpvNG2^+^ cells into osteogenic cells (a), adipocytes (b), cartilage cells (c). (**F**a–c) MLpvNG2^+^ cell proliferation detected by CCk-8 assay confirmed the changed in morphology from (a–b) and fast response (10 min) (c) of MLpvNG2^+^ cells to pathological signals from homogenate of cirrhotic liver induced by DEN (Cir-hm). Data are shown as means ± standard error of the mean (SEM) of duplicate preparations from three independent experiments. Scale bars = 100 μm (**B,D,F**). Scale bars = 50 μm (**A,E**)

After isolation, cell colonies began to emerge after 3 weeks (Fig. 1Aa). Freshly isolated cells (P0) grew slow and had only a few cells after 30 days (Fig. 1Ab); cells reached 60% confluence at 40 days (Fig. 1Ac). These cells initially had a characteristic morphology with prominent nuclei and relatively limited perinuclear cytoplasm^28^,^29^ (Fig. 1Ae,Da). Most of the P1 (not shown) and P2 cells assumed a rhomboid morphology and grew to 60% confluence within 10 days (Fig. 1Ad). By labeled culture cells with NG2 antibody, 95% of the cells were NG2 positive (Fig. 1Ae), 7% of NG2-expressing cells were co-labeled with CK19, 78% with Sca-1, 90% with CD133, 83% with DLK and 78% with PDGFR-β (Fig. 1B). Flow cytometry revealed that NG2-expressing cells co-labeled with EpCAM, CD14, CD24, and CD49f (Fig. 1Ca–d) suggesting their hepacyte progenitors^30^. Colony formation assay showed that within 10 days culture, the number of single NG2-expressing cells growing into colonies gradually increased (Supplementary Fig. 1), suggesting every NG2-expressing cell in the population for its ability to undergo “unlimited” division. By contrast, <0.5% of the NG2-expressing cells were positive for vWf (von Willebrand factor) (Fig. 1Ce), CD34 and CD45 (not shown), namely MLpvNG2^+^ cells (mouse liver periportal vascular region NG2-expression cells) in this study. To determine whether the phenotype and fundamental properties of MLpvNG2^+^ cells changed during long-term culture (as has been reported for progenitors^31^, cell morphology and numbers from P2 to P8 were compared with respect to proliferation rate. The morphology was similar for P2, P4 and P8 cells that strongly expressed NG2 (Fig. 1Da–c). BrdU (5-Bromo-2-deoxyUridine) incorporation analysis demonstrated that P2, P4 and P8 cells possessed a similar rate of proliferation (Fig. 1Dd) suggesting that the isolated MLpvNG2^+^ remained undifferentiated after 8 passages. In addition, α-SMA and Oct4, two markers of non-differentiation^32^,^33^, were also tested to confirm the non-differentiation state of these cells (not shown).

MLpvNG2^+^ cells have the capacity to induce trilineage differentiation including adipocytes (Fig. 1Ea), osteocytes (Fig. 1Eb) and chondrocytes (Fig. 1Ec), as detected by oil Red O, alizarin red and alcian blue, respectively. Interestingly, MLpvNG2^+^ cells response to cirrhotic signals within 10 min showed changes in morphology from sheet shape in physiological condition (Nai-hm) (Fig. 1Fa), to round or oval shape (Fig. 1Fb indicated as arrows). Similarly, fast response of MLpvNG2^+^ cells to Cir-p-hm in cell growth rate was also detected by using CCK-8 assay (Fig. 1Fc)^34^, suggesting emergency repair potential of MLpvNG2^+^ cells in pathological conditions. Take together, our isolation procedure yielded MLpvNG2^+^ cells may be a novel adult mouse liver stem cell population that have some unidentified different biological and functional features.

### MLpvNG2^+^ cell administration reduces both fibrogenesis and hepatic stellate cell activation and enhances functional proteins in a diethylnitrosamine (DEN)-induced liver cirrhotic mouse model

**Figure 2:**
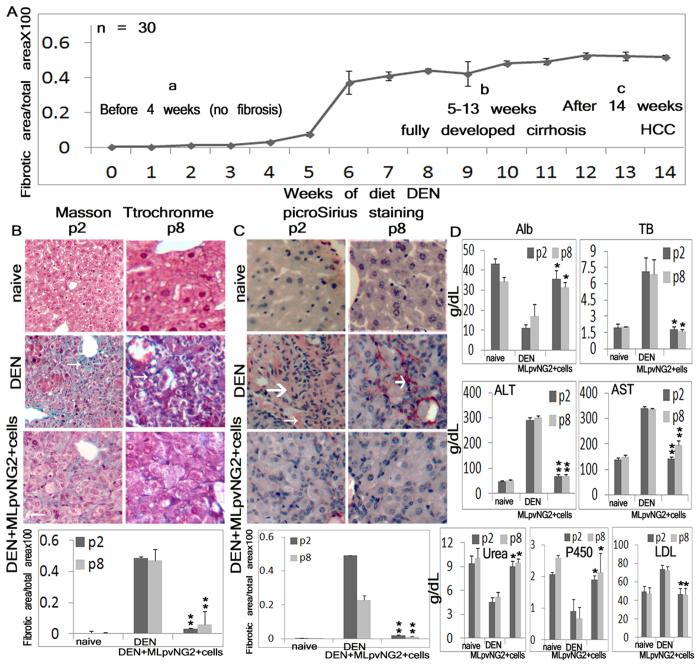
Improvement in cirrhosis and hepatic functionality following MLpvNG2^+^ cell treatment. (**A**a–c) Induction of liver cirrhosis by DEN from 1–4 weeks (a), 5–13 weeks (b) and 14 weeks (c) (n = 30). **(B,C)** The blue color of Masson Trichrome method stained for collagen fibers (**B**) indicated as thin arrows) and the red color of picroSirius Red method stained for reticular fibres (**C**) indicated as small wide arrow), the basal laminae of capillaries (**C**) indicated as large wide arrow) and co-aligned molecules of Type I collagen (**C**) indicated as tiny thin arrow) after P2 and P8 MLpvNG2^+^ cell treatment (n = 11). Scale bar = 50μm, Summarized data assessing levels of Alb, Total bilirubin (TB), AST, ALT, urea, CytP450 and LDL in serum obtained from naïve animals (P2, n = 14; P8, n = 9) and animals treated with DEN (P2, n = 10; P8, n = 10) and DEN plus MLpvNG2^+^ cells (P2, n = 9; P8, n = 7) by ELISA in (**D**). Data are shown as mean ± SEM of duplicate preparations from 3–10 independent experiments. *P < 0.05, **P < 0.01 DEN^+^ MLpvNG2^+^ cell treated mice *vs.* DEN^+^ PBS treated mice.

To determine if MLpvNG2^+^ cells promote repair in the setting of liver cirrhosis, the functional effects of intravenous infusion of MLpvNG2^+^ (both P2 and P8) were evaluated in mice with ongoing DEN-induced liver cirrhosis^35^. DEN induces microscopic fibrosis in the liver as revealed by positive staining with Masson’s Trichrome^36^ (Fig. 2A). Prior to 4 weeks, there was no fibrosis in the liver (Fig. 2Aa). DEN resulted in a progressive increase in fibrosis of the liver by week 5–9 and was fully developed by week 10–13 (Fig. 2Ab). Hepatocellular carcinoma cells (HCC) can be identified at 14 weeks (Fig. 2Ac) by H&E staining^37^ (not shown). Livers treated with MLpvNG2^+^ for 4 weeks (starting at 6 weeks of DEN treatment) showed similar results between P2 and P8 using Masson’s Trichrome and picroSirius staining. Using Masson’s Trichrome staining (Fig. 2B) (n = 6), livers from MLpvNG2^+^ cell-treated mice showed a 13.4-fold (P2) and 8.2-fold (P8) reduction in DEN-induced fibrosis compared with PBS-treated animals (P2: 3.1 ± 1.3% *vs.* 48.4 ± 1.3%; P8: 5.7 ± 1.4% *vs.* 47.0 ± 1.7%) (*P* < 0.01). A similar result was observed using picroSirius staining (Fig. 2C) (n = 6), suggesting that MLpvNG2^+^ cells exhibited similar functional activities between P2 and P8 in improving inflammation and fibrogenesis. Serum was collected from animals and assayed for Albumin (Alb), Total Bilirubin (TB), alanine aminotransferase (ALT), aspartate aminotransferase (AST), urea (URE), cytochrome P450 (CytP450) and low density lipoprotein (LDL) (Fig. 2D). Similar effect on restoring those levels was detected in P2 and P8 cell-treated mice. In the DEN + MLpvNG2^+^ cells group, Alb and TB levels were substantially improved (*P* < 0.05). The increase in ALT and AST activities were significantly attenuated in the DEN + MLpvNG2^+^ cells group (*P* < 0.01). Finally, DEN-disrupted liver expression of URE, CytP450 and LDL were also improved by MLpvNG2^+^ cells treatment (*P* < 0.05). Given the lack of evident differences in the cell population up to P8, all subsequent studies were conducted in MLpvNG2^+^ cells at P8 or lower passages.

### Reduction of immune response and hepatic stellate cells after MLpvNG2^+^ cells transplantation

**Figure 3:**
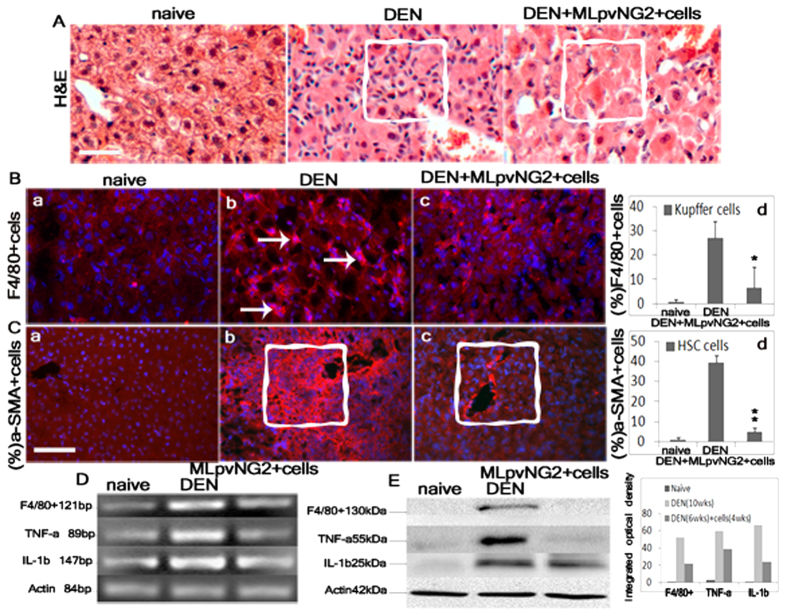
Decrease of mononuclear cell infiltration, activation of hepatic stellate cells (HSC) and inflammation after MLpvNG2^+^ cell transplantation. (**A**) H&E staining of naïve, DEN-induced cirrhotic and DEN-induced cirrhotic liver plus MLpvNG2^+^ cell treatment, infiltrating cells indicated within squares (n = 5). Scale bar = 50μm. Immunohistochemistry depicting the presence of F4/80^+^ kupffer cells (**B**a–c), F4/80^+^ cells indicated as arrows) (n = 6), α-SMA^+^ HSC (n = 8) (**C**a–c) in naive liver (a), DEN-diet liver (b) and DNE plus cell treatment liver (c), α-SMA^+^ cells indicated as squares. Scale bar = 100 μm. (**B**d,**C**d) Summarized data depicting the % of positive staining within the total cells. Representative blots depicting the level of mRNA expression by RT-PCR(n = 3) (**D**) and protein expression by western blot analysis (n = 3) (**E**) for F4^+^/80, TNFα and IL-1β in naïve, DEN-induced cirrhotic liver and DEN-induced cirrhotic liver plus MLpvNG2^+^ cell treatment. DAPI (blue) staining was used to visualize nuclei. Data are shown as mean ± SEM. *P < 0.05, **P < 0.01 DEN^+^ MLpvNG2^+^ cell treated mice *vs.* DEN^+^ PBS treated mice.

Sections of liver from animals with liver cirrhosis were stained with H&E to identify mononuclear cells, labeled with anti-F4/80^+^, anti-α-SMA antibodies to identify a subset of inflammatory resident macrophages (Kupffer cells, KCs)^38^ and hepatic stellate cells (HSC). qRT-PCR and western blot assays were used to identify gene expressions of F4/80^+^, TNF-α and IL-1b (Fig. 3). H&E staining showed that the numbers of infiltrating cells (indicated within squares) was substantially attenuated following MLpvNG2^+^ cells treatment (Fig. 3A). Immunofluorescence demonstrated that DEN caused a significant increase in the number of F4/80^+^ cells in the liver of mice receiving PBS (27.7 ± 2.4%) (Fig. 3Bb), which was significantly reduced with MLpvNG2^+^ cells treatment (7.2 ± 3.7%) (Fig. 3Bc) (P < 0.05) but higher than controls (0.5 ± 0.3%) (Fig. 3Ba) (n = 6). The number of F4/80^+^ cells was reduced ~3.7-fold in animals treated with MLpvNG2^+^ cells compared with PBS-treated controls (Fig. 3Bd) (*p* < *0.05*), suggesting F4/80^+^ KCs participating in immunoinflammatory reactions^38^. The number of α-SMA^+^ HSC was markedly reduced in the liver of mice treated with MLpvNG2^+^ cells (Fig. 3Cc) compared with those in PBS-treated animals (Fig. 3Cb, indicated within squares) (*P* < *0.01*), which was similar to that observed in controls (Fig. 3Ca). In addition, reduction of TNF-α and IL-1β gene expressions after MLpvNG2^+^ cells transplantation was verified by qRT-PCR (Fig. 3D) and western blot (Fig. 3E), suggesting that the relieved fibrotic load and restored functions of MLpvNG2^+^ cells in mice with cirrhotic livers might involve mononuclear cells, Kupffer cells, HSC, TNF-α and IL-1β.

### MLpvNG2^+^ cells home to the liver and differentiate directly into hepatocyte lineages

**Figure 4:**
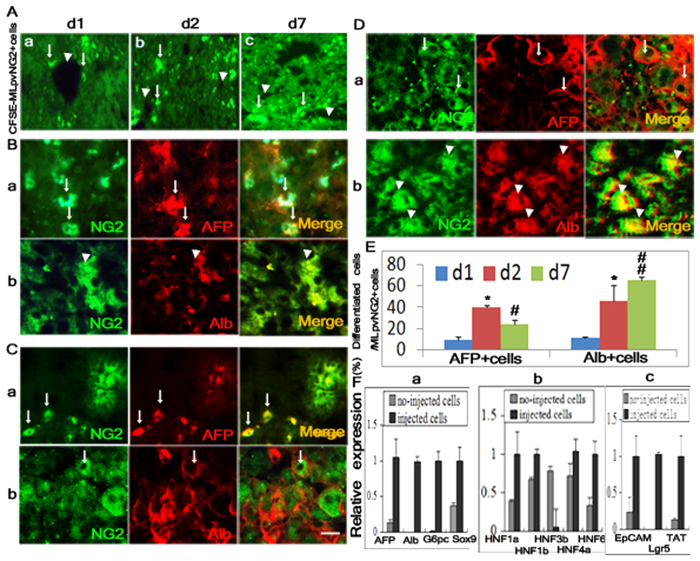
Migration and hepatocyte differentiation of MLpvNG2^+^ cells in liver cirrhosis. (**A**a–c) Engrafted MLpvNG2^+^ cells labeled by CFSE (green) appeared around blood vessels in DEN-induced cirrhotic liver at 1 d (a), 2 d (b) and 7 d (c) (grafted cells indicated as arrows; vessels indicated as arrowheads). Immunocytochemistry of engrafted CFSE-labeled MLpvNG2^+^ cells differentiated into alpha fetal protein (AFP^+^) hepatic stem/progenitors (a, shown as arrows) and Alb^+^ cells (b, shown as arrowheads) at 1d (**B**), 2 d (**C**), and 7 d (**D**). (**E**) Summarized data depicting the % AFP^+^ and Alb^+^ differentiation *in vivo* (n = 6). (**F**) CFSE-labeled MLpvNG2^+^ cells was isolated from the DEN-induced cirrhotic plus MLpvNG2^+^ cell treatment liver by FACS sorting on d7 and relative mRNA expression levels of various hepatic developmental associated genes against un-transplanted MLpvNG2^+^ cells were detected by qRT-PCR (n = 3). *P < 0.05, AFP: d1 compared with d2; d1 compared with d7; d2 compared with d7 (reduced AFP number). **P < 0.01, Alb: d1 compared with d2; d1 compared with d7; d2 compared with d7 (increased Alb number). P values also refer to Panel (**E**) and/or (**F**). Data are shown as mean ± SEM of duplicate preparations from 6 independent experiments. Scale bar = 100 μm.

We found that at 1 day following injection of MLpvNG2^+^ cells, some Carboxyfluorescein succinimidyl ester (CFSE)-labeled MLpvNG2^+^ around blood vessels in the recipient liver (Fig. 4Aa), were increased at 2 days (Fig. 4Ab). By day 7, except for surrounding blood vessels (Fig. 4Ac, arrowheads indicated as vessels and arrows indicated as migrated cells), numerous labeled cells were found to be distributed in the liver and formed colonies. Immunohistochemistry showed that by day 1 following transplantation, 9.1 ± 2.9% of CFSE-labeled MLpvNG2^+^ cells differentiated into AFP^+^ cells (red) (Fig. 4Ba) and 11.2 ± 0.8% of MLpvNG2^+^ cells differentiated into Alb^+^ cells (red) (Fig. 4Bb) in the liver. By day 2, numbers were increased to 39.4 ± 1.7% of differentiated AFP^+^ (Fig. 4Ca) and 45.6 ± 4.8% of Alb^+^ cells (red) (Fig. 4Cb). By day 7, 23.6 ± 3.9% of the MLpvNG2^+^ cells differentiated into AFP^+^ cells (red) (Fig. 4Da) and 65.4 ± 2.7% into Alb^+^ cells (red) (Fig. 4Db) in the liver. Figure 4E shows the summarized quantitative data for differentiated AFP^+^ and Alb^+^ cells on days 1, 2 and 7. To assess hepatic gene expression within DEN liver, CFSE-labeled MLpvNG2^+^ cells were isolated by FACS on day 7 and the hepatic associated genes were detected by qRT-PCR. The gene expression in CFSE-labeled MLpvNG2^+^ cells group was significantly higher than that of non-injected MLpvNG2^+^ cells including hepatic functional genes of AFP, ALB, glucose-6-phosphatase (G6Pc), Sox9 (Fig. 4Fa), hepatic nuclear factor genes of HNF1α/β, HNF3β, HNF4α, HNF6 (Fig. 4Fb) and bile duct genes of EpCAM, Lgr5, tyrosine aminotransferase (TAT) (Fig. 4Fc). These results suggest that the MLpvNG2^+^ cells have the capacity to differentiate into the hepatocyte lineage in the recipient liver.

### Conversion of MLpvNG2^+^ cells into hepatic lineages *in vitro* in response to liver cirrhosis-induced cues

**Figure 5:**
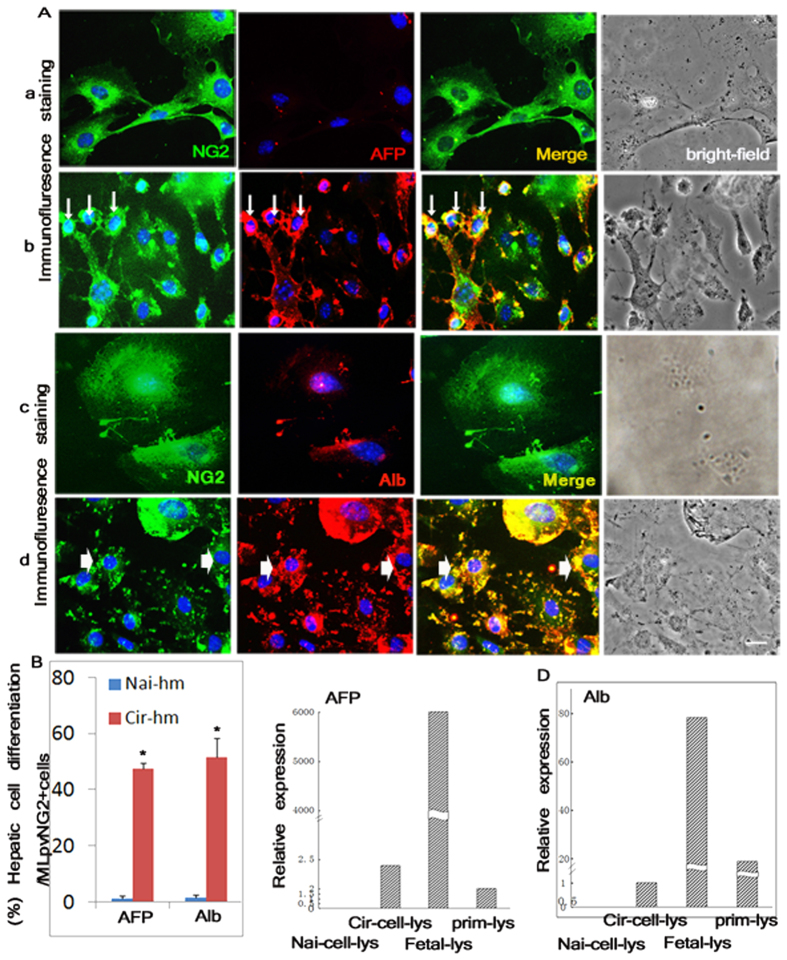
Hepatic lineage differentiations of MLpvNG2^+^ cells in response to cirrhotic cues. (**A**a–d) Immunohistochemistry of hepatic cell differentiation of MLpvNG2^+^ cells in naïve liver homogenate AFP^+^ cells (a), Alb^+^ cells (c) and in cirrhotic liver homogenate AFP^+^ cells (b), differentiated AFP^+^ cells indicated as thin arrows), Alb^+^ cells (d), differentiated Alb^+^ cells indicated as wide arrows). DAPI (blue) staining was used to visualize nuclei. **(B)** Summarized data for A comparing % differentiation of AFP and Alb positive cells obtained from naïve (Nai-hm) and DEN-diet treated liver (Cir-hm) homogenates (n = 5). Summarized data of comparing mRNA expression levels of AFP (**C**) and Alb (**D**) in the groups of cell lysates obtained from primary hepatocytes (Prim-lys), the cell lyses obtained from recipient liver treated with MLpvNG2^+^ cells (Cir-cells-lys), the cell lyses obtained from the healthy liver without treatment (Nai-cell-lys) and the cell lysates that obtained from fetal livers (fetal-lys) as assessed by qRT-PCR. Scale bar = 100 μm. Data are shown as mean ± SEM, **P < 0.01 *vs.* Nai-hm.

To address the functional hepatic cell differentiation-potential in response to cirrhotic signals, First, MLpvNG2^+^ cells were plated at low-density (1 × 10^5^) in the presence of Cir-hm and the number of hepatic lineage cells that developed was compared with cultures grown in Nai-hm. By 1 h, MLpvNG2^+^ cells cultured in Cir-hm were markedly activated for AFP^+^ and Alb^+^ hepatic cell differentiation. AFP^+^ and Alb^+^ cells constituted 47.3 ± 2.1% (Fig. 5Ab) and 51.4 ± 7.0% (Fig 5Ad), respectively, and 10.04 ± 1.9% of CK19^+^ cells, a bile duct marker (Supplementary Fig. 2 arrows indicate CK19^+^ cells), from the total MLpvNG2^+^ cells (*P* < 0.05), suggesting that MLpvNG2^+^ cells possess a substantial capacity to differentiate rapidly into hepatic stem cells, mature hepatocytes and bile duct cells^39^ in response to pathological stimuli. No AFP^+^ (Fig. 5Aa), Alb^+^ (Fig. 5Ac) or CK19^+^ (not shown) cells could be detected in Nai-hm, suggesting that MLpvNG2^+^ cells remain quiescent under normal conditions. Figure 5B depicts the summarized data from Fig. 5Ab,d for the percent differentiation of hepatic lineage for the AFP^+^ and Alb^+^ cells from cultured MLpvNG2^+^ cells (*P* < 0.05). To support the evidence of hepatic differentiation of MLpvNG2^+^ cells in a pathological stimulus, further tests were confirmed for AFP (Fig. 5C) and Alb (Fig. 5D) by qRT-PCR analysis and compared with that observed in fetal (fetal-lys) and primary hepatocytes (Prim-lys). Similar results to that of hepatic cell differentiation of MLpvNG2^+^ cells in response to cirrhotic signals were found. Relative gene expression for AFP and Alb was found significantly higher in the MLpvNG2^+^ cells cultured in Cir-hm (Cir-cell-lys) compared with cells cultured in Nai-hm (Nai-cell-lys) (AFP: 0.0008 ± 1.48 *vs.* 2.1734 ± 0.08, *P* < 0.0001; Alb: 1.15 ± 0.0000 *vs*. 1.0000 ± 0.93, *P* < 0.0001). These observations indicate that the MLpvNG2^+^ cells could be the population that actively differentiates into cells along the hepatocyte lineage in response to injury signals.

### MLpvNG2^+^ cell signals impact on endogenous stem/progenitors and play a protagonist role on the improvement of functions in the mice with DEN-induced liver cirrhosis

**Figure 6:**
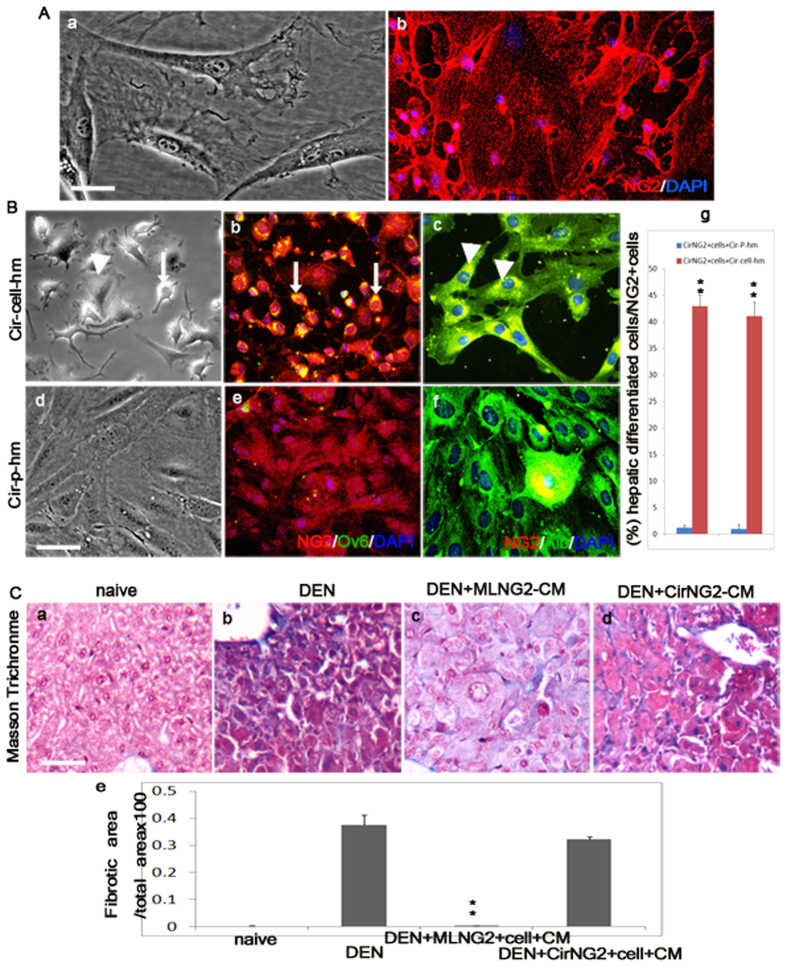
CirNG2^+^ cells response to grafted MLpvNG2^+^ cell signals. NG2-expressing cells were isolated from liver of mice that had been administered DEN for 6 weeks (designated CirNG2^+^ cells) by using same procedure as used for MLpvNG2^+^ cells. (**A**) (a,b) Cultured CirNG2^+^ cells (**a**) and stained with antibody against NG2 (**b**). Immunocytochemistry of differentiated OV6^+^ and Alb^+^ cells from CirNG2^+^ cells cultured in Cir-cell-hm **(B**a–c, g) and Cir-P-hm ( = Cir-PBS-hm) (**B**d–g) (n = 3). DAPI (blue) staining was used to visualize nuclei. **P < 0.01 *vs.* Cir-P-hm. Conditioned medium was then collected from cultured MLpvNG2^+^ cells (MLNG2-CM) and CirNG2^+^ cells (CirNG2-CM) and injected into DEN-induced liver cirrhosis model. Four weeks after injection, liver sections from all treated groups were stained with Masson for fibrogenesis detection. (**C**a–d) Masson staining for fibrosis in the livers of naïve (a), DEN (b), DEN plus MLNG2-CM (c) and DEN plus CirNG2-CM (d). (**C**e) Summarized data depicting the analysis from Ca-d (n = 4). Scale bar = 100 μm. Data are shown as mean ± SEM, **P < 0.01 DEN^+^ MLNG2-CM *vs.* DEN^+^ CirNG2-CM.

NG2-expressing stem/progenitors were isolated from cirrhotic liver of mice that had been administered DEN for 6 weeks by using the same procedure as used for MLpvNG2^+^ cells, namely CirNG2^+^ cells (Fig. 6Aa), and stained with anti-NG2 antibody (Fig. 6Ab). CirNG2^+^ cells were cultured with liver tissue homogenate prepared from DEN + MLpvNG2^+^ cells (Cir-cell-hm) and compared with the liver tissue homogenate prepared from DEN + PPBS mice (Cir-PBS-hm). By 24 h, morphological changes in CirNG2^+^ cells were observed to be toward a more oval (Fig. 6Ba, arrow) and flat-round (Fig. 6Ba, arrowhead) appearance. When tagged with antibodies against Ov6 and Alb, strong expression of Ov6 (Fig. 6Bb arrows) and Alb (Fig. 6Bc arrowheads) in these CirNG2^+^ cells was observed. In contrast, CirNG2^+^ cells maintained their original morphology when cultured with Cir-PBS-hm (Fig. 6Bd) and no Ov6^+^ and a few (less than 1%) Alb^+^ cells were detected (Fig. 6Be,f), suggesting that grafted MLpvNG2^+^ cells could release some unknown signals to promote the hepatic cell differentiation of endogenous liver stem cells. Ov6 and Alb protein expression levels in CirNG2^+^ cells cultured in the Cir-cell-hm were higher than those in the CirNG2^+^ cells cultured in the Cir-PBS-hm (both *P* < 0.01) (Fig. 6Bg).

Conditioned medium from cultured MLpvNG2^+^ cells (MLNG2-CM) and CirNG2^+^ cells (CirNG2-CM) were collected, and injected into DEN-induced liver cirrhosis models. By 4 weeks after injection, liver sections from all treated groups were stained with Masson for fibrogenesis detection (Fig. 6C). Mice treated with MLNG2-CM showed reduction in fibrosis, which was 0.30 ± 0.01% (Fig. 6Cc), compared with CirNG2-CM treated animals (32.1 ± 1.1%) (Fig. 6Cd) (*P* < 0.01), and to the DEN group (27.4 ± 3.9%) (Fig. 6Cb). Although MLNG2-CM-treated livers were not recovered like normal livers (Fig. 6Ca,e), treatment with MLNG2-CM results in higher recovery of hepatic function compared with CirNG2-CM. These observations indicated that grafted MLNG2-CM administration plays a major role on improvement of injured liver functions.

## Discussion

Liver transplantation remains the last therapeutic option for chronic liver diseases such as liver cirrhosis. However, a major obstacle to further development of organ transplantation is the shortage of available donor organs. An obvious and broadly applicable option would be the transplantation of liver cells. The ability to isolate distinct cell populations and characterize their functional properties will lead to the identification of the mechanisms mediating repair and the cell populations appropriate for the treatment of chronic and acute liver diseases. Here, a novel approach was described for the isolation of a population of undifferentiated cells from the healthy adult mouse liver periportal vascular region through the “Percoll-Plate-Wait” procedure. The cell population was isolated through a dissociation and Percoll gradient selection process that resulted in a relatively homogenous population of cells that expressed the cell surface glycoprotein NG2^15^. These isolated NG2-expressing cells (MLpvNG2^+^ cells), from genetically normal mice, were highly expendably and phenotypically stable *in vitro*. MLpvNG2^+^ cells co-expressed with stem cell markers of Sca-1, Cd133, DLK)^30^, and liver stem/progenitor markers of EpCAM, CD14, CD24, CD49f)^40^, but few expressed with hematopoietic and endothelial markers of CD31, CD45 and vWf^41^,^42^, suggesting that these cells are liver progenitores^39^. When infused into mice with ongoing DEN-induced liver cirrhosis, these cells promoted functional recovery that was associated with a reduction in the extent of fibrotic lesions, stimulation of endogenous liver progenitors, and lower levels of F4/80^+^ KC and α-SMA^+^ HSC infiltration. Exposure of MLpvNG2^+^ cells to medium conditioned by cirrhotic liver homogenate derived from the livers of animals with liver cirrhosis, stimulated the generations of hepatic lineages (AFP^+^, Alb^+^, CK19^+^) suggesting that fate determination in these NG2-expressing hepatic progenitors can be modulated by pathologically-derived signals that may highlight their *in vivo* hepatic lineage differentiation potency^43^. Therefore, MLpvNG2^+^ cells would be a potential future alternative to functional hepatocytes or liver transplantation for liver cirrhosis.

The *in vivo* correlate of the isolated MLpvNG2^+^ cells is currently unclear, although they co-express with PDGFR-β, a typical mouse MSC marker^44^, but relatively few with hepatic oval cells^45^. MLpvNG2^+^ cells do not express CD34^+^ or A6 which are markers of hepatic oval cells (HOC) in adult mouse liver^46^. First, HOC can be identified by the expression of CD34^31^, a lineage-specific marker to identify hematopoietic stem cells^47^ but not NG2. Second, the morphology of the cells was not consistent with that of HOCs, which present oval shape but seldom assume a flattened sheet shape morphology such as that observed in MLpvNG2^+^ cells. An unclear aspect of MLpvNG2^+^ cells is their ability to generate multiple cell types. In the present study, the ability of MLpvNG2^+^ cells to differentiate into adipocytes, chondrocytes and osteocytes suggested their possible mesodermal origin^48^ and their multipotency. In our case, FACS analysis identified that less than 1% of NG2-positive cells reside in liver periportal vascular region (not shown), and immunofluorescence staining demonstrated that isolated MLpvNG2^+^ cells share some characteristics with hepatic stem/progenitors^40^. Whether MLpvNG2^+^ cell is a progenitor for the elusive oval cells and BM-MSCs need to be further delineated. However, based on our recent studies, we define MLpvNG2^+^ cells as a liver stem cells that is different from MSCs because of their absence of Sox2 gene expression (unpublished work) which MSCs does^49^. MLpvNG2^+^ cells may not be oval cells either but a novel liver stem/progenitors that might be different from reported hepatic progenitors (HPC)^50^. Although the concept of HPC and HOC in this field of study is still controversial^51^,^52^, MLpvNG2^+^ cells could rapidly generate functional (Alb^+^) hepatocytes in response to signals of diseased livers which is not been described for HPCs, or HOC^53^,^54^,^55^,^56^,^57^,^58^. In addition, MLpvNg2^+^ cells were found not to express CD34 and A6, the specific markers for HOC^45^. Recent studies have shown that bile duct (BD) was found to generate hepatocytes^59^,^60^. NG2-expressing cells do not express in the BD region (unpublished work), suggesting the differences are not only between the MLpvNG2^+^ cell, MSC, HOC but BD cell as well (Supplementary Table, with references).

Another characteristic shared by MLpvNG2^+^ cells and stem cell populations is the ability to promote functional recovery in animal models of liver cirrhosis. To address whether MLpvNG2^+^ cells could play a role in tissue regeneration and/or repair of tissue injury in the host organ system, the DEN-induced murine liver cirrhosis model was used^61^,^62^. Tracing results from infused CFSE-labeled MLpvNG2^+^ cells^62^ in the DEN-induced ongoing liver cirrhotic mouse model indicated that MLpvNG2^+^ cells had migrated into cirrhotic liver and promoted functional recovery, which was associated with a reduction in the extent of fibrotic lesions, mononuclear cells, number of Kupffer cells, and number of HSCs; restoration of functional liver proteins; differentiation into hepatic lineage including AFP- and Alb-expressing cells; hepatic-specific transcription factors (HNF1, HNF4α, HNF6, and HNF3β; liver-associated enzymes of aminotransferase (TAT), glucose-6-phosphatase-α (G6Pase-α or G6PC) and EpCAM^63^,^64^,^65^; and promotion of endogenous progenitor cells to join repair. These results suggest that the cirrhotic liver-derived signals have independent inductive potential for the direct differentiation of MLpvNG2^+^ cells into the hepatic lineage in response to injury signals resulting in promoting functional recovery in animal models of liver cirrhosis^66^,^67^. Therefore, MLpvNG2^+^ cells may be a good source for both liver stem cells and functional hepatocytes in response to pathological signals.

Previous studies have demonstrated that treatment with MSCs derived from either human or mouse sources promoted functional recovery in the liver cirrhosis model^68^,^69^. Similarly, hepatic progenitor cells promote functional recovery in liver cirrhosis, suggesting that this is a common characteristic of stem/progenitors^70^. In both cases, modulation of the immune response is associated with the promotion of repair. A clear function of MSCs is to promote a switch in the immune response from a Th1-based pro-inflammatory to a Th2 anti-inflammatory response^71^. However, the contribution of this switch to the overall functional improvement in liver diseases is not yet defined.

The specific mechanisms by which MLpvNG2^+^ cells promote recovery in the DEN-induced liver cirrhosis model is unclear, but may be closely tied to the process of functional recovery in the intact adult liver. Several lines of evidence support our hypothesis that the effect of MLpvNG2^+^ cells on liver tissue repair involves more than just differentiation into hepatic cell types in response to signals from the injured liver, since grafted MLpvNG2^+^ cells would provide only less than 0.5% of the estimated total functional hepatocyte population in the recipient liver. The MLpvNG2^+^ cells also seem to mediate a reduced immune response (F4/80^+^ Kupffer cells), enhanced function, fibrotic improvement in reduction of the number in α-SMS^+^ HSC, stimulation of endogenous repair and regeneration of DEN-induced liver cirrhosis. The reduction in Kupfer cells seen in the lesions of animals treated with MLpvNG2^+^ cells suggests that they may directly influence the immune system. Alternatively, the reduction in the number of HSC, restoration of liver markers^72^,^73^,^74^ and histological improvement may reflect a primary contribution of the MLpvNG2^+^ cells to tissue repair and regeneration. Inhibition of inflammatory reaction and switching the intrahepatic environment from pro-fibrogenesis to pro-hepatocytogenesis by engrafted healthy MLpvNG2^+^ cells may play a pivotal role in mobilizing endogenous machinery for repair.

Promotion of hepatic differentiation (AFP^+^ and Alb^+^) of CirNG2^+^ cells after healthy MLpvNG2^+^ cells transplantation in the setting of liver cirrhosis suggest that MLpvNG2^+^ cells have the capacity to mobilize endogenous stem/progenitors and promote their differentiation into liver cells for tissue repair. This differentiation of CirNG2^+^ cells in response to signals released from the liver of animals with ongoing cirrhosis treated with MLpvNG2^+^ cells (Cir-cell-hm) suggests that MLpvNG2^+^ cells could stimulate endogenous repair by mobilizing endogenous liver stem cells. The findings that injection of the conditioned medium (CM) collected from cultured MLpvNG2^+^ cells (MLNG2-CM) dramatically promoted endogenous CirNG2^+^ cell hepatic cell differentiation (Fig. 6Ba–c,g) and significantly reduced fibrogenesis (Fig. 6Cc,e) comparing to the injection of the CM collected from cultured CirNG2^+^ cells (CirNG2-CM) (Fig. 6Bd–f,g,Cd,e) suggest that MLpvNG2^+^ cells serve as a protagonist in the improvement of hepatocyte functions in DEN-induced liver cirrhosis. In addition, in the setting of liver cirrhosis, MLpvNG2^+^ cells may reside in areas of injured livers and differentiate into hepatic cells that contribute to injured liver repair. Consistent with this hypothesis are the findings of the *in vitro* co-culture systems in the present study: MLpvNG2^+^ cells differentiated into immature and mature hepatocytes in response to DEN-induced liver cirrhosis signals released from the liver of animals with ongoing cirrhosis. In normal conditions, they might be recruited for turnover and replacement functions that are upregulated in response to injury signals.

The nature of the signals that mediate enhanced host repair and mature hepatocyte numbers in response to MLpvNG2^+^ cells infiltration is currently unknown but may include growth factors such as HGF, PDGF, and IGF-1^75^,^76^,^77^. Likewise, whether MLpvNG2^+^ cells promote hepatocytes survival from MLpvNG2^+^ cells or the existing hepatocytes will require additional analysis. The signals that promote the responses of MLpvNG2^+^ cells to liver cirrhotic cues are also currently unknown and their identification may reveal important targets for future therapeutic approaches to liver cirrhosis. The present study suggests that the microenvironment in liver lesions such as those in liver cirrhosis exerts an influence on MLpvNG2^+^ cell differentiation, and CirNG2^+^ cells can be stimulated by extracellular signals from MLpvNG2^+^ cells, while conversion to CirNG2^+^ cells by MLpvNG2^+^ cells could provide support for repair in the liver. The evidence for HSC derived from MLpvNG2^+^ cells was not detected in this study and need further analysis. The present study is limited by its *in vivo* nature. In addition, the MLpvNG2^+^ cells require further characterization. As such,, further study is needed before clinical trials.

In conclusion, a novel Percoll-Plate-Waiting approach allowed the isolation of a population of cells from adult mouse liver portal perivascular regions that promotes functional recovery in DEN-induced liver cirrhosis models and regeneration in liver cirrhosis. These cells express NG2 and PDGFR-β, also normal stem and hepatic progenitor markers. Treatment of animals with ongoing DEN-induced liver cirrhosis with MLpvNG2^+^ cells resulted in rapid infiltration (24 h) of the cells into the livers and significant functional improvement. This improvement was correlated with enhanced generation of host immature and mature hepatic cells as well as the conversion of endogenous progenitors (CirNG2^+^ cells) to hepatocytes. Given that MLpvNG2^+^ cells are isolated in relatively small numbers that are generated from adult liver tissue, they are unlikely to be a direct source for cell therapy in the immediate future. Further analysis of their origin, and the mechanisms underlying their ability to promote tissue repair in liver cirrhosis will provide valuable insights into the cellular and molecular pathways that mediate recovery from liver diseases. Such data are fundamentally important to the future development of novel therapies for liver repair in liver diseases such as liver cirrhosis.

## Methods

### Animals

C57BL/6 J male adult mice (10–12 weeks old, 22–24 g) were purchased from the Third Military Medical University, Chongqing, China. Mice were bred and maintained in an air-conditioned animal center with specific pathogen-free conditions, a 12-h cycle of daylight, and unlimited access to chow and water until 2 weeks before the experiment. Experimental protocols were approved by the Institutional Animal Care and Use Committee of the Third Military Medical University (#SYXK YU 2012–00 12). The methods were carried out in accordance with the approved guidelines.

### Isolation of MLpvNG2^+^ cells using “Percoll-Plate-Wait” procedure

MLpvNG2^+^ cells were isolated based on published methods^32^. Step 1: Percoll gradient isolation. Mice (10–12 weeks old) were sacrificed by cervical dislocation. The carcass was sprayed liberally with 70% (vol/vol) ethanol. The liver periportal vascular regions were cut (2/mice) by using surgical scalpel. Any attached parenchymal tissues was pealed from the resected tissue blocks. The two tissue blocks were pooled (~0.5 g) (Supplementary Fig. 3A,B). A single cell suspension was obtained in cold minimum essential medium (MEM) (without Ca^2+^ and Mg^2+^, wMEM) (Hyclone, SH30265.01), and digested in 3 mL of trypsin (0.05%)/EDTA (0.02%) (Hyclone, SH30042.01) at 37 °C with gentle agitation in 5% CO_2_ for 30 min (Supplementary Fig. 3C). Digested tissues were then mixed with 0.6 mL of 0.5% DNase I (Sigma, AMPD1) in wMEM containing 10% heat-inactivated fetal bovine serum (FBS) (Sigma, A-7906) at 37 °C with gentle agitation in 5% CO_2_ for 10 min. Red blood cells were lyzed using 1 mL of ice-cold sterile H_2_O for 6 seconds. Immediately after lysis, 1 mL of 2× PBS with 4% (vol/vol) FBS was added to quench the reaction. Volume was completed to 15 mL with wMEM immediately after^58^. Cells were filtered through a 70-μm cell strainer on the top of a 50-mL conical tube, and then centrifuged at 1,200 rpm for 5 min. Cells were washed twice before layering onto a Percoll gradient (GE Healthcare, 10055500). The gradient was prepared from stock isotonic Percoll (SIP) at 70% in 1× PBS, 30% in 1× wMEM. Cells were homogenized in 7.5 mL of 30% SIP and slowly layered on top of the 7.5 mL 70% SIP. Following centrifugation at 2,000 rpm for 30 min, 1.0–2.0 mL of cell fraction of the 70–30% interface was collected into a clean conical tube and washed at 1,200 rpm for 5 min. Supernatant was aspirated and the pellet was resuspended by vigorous agitation until the cell suspension was homogenous.

Step 2: to plate cells. Cells (1 × 10^6^) in 3 mL of DMEM/F12 medium (Gibco-BRL, 11330) containing 10% FBS and 1% antibiotic-antimycotic solution (Cellgrow, 30-001-CI) (complete medium) were plated in poly-L-lysine (PLL) (Sigma, P1399)-24 h coated 75-cm^2^ flasks (Thermo, 156472) in DMEM/F12 culture medium containing 10% FBS (complete medium) at 37 °C in 5% CO_2_ for 20 min. An additional 22 mL of complete medium was added for starting primary culture.

Step 3: waiting procedure. In the first three weeks of primary culture, only one mL of complete medium was added once a week until NG2^+^ colonies appeared (at day 22). After colony appearance, one mL of complete medium was added every 3 days until 6 weeks or until the cells reached about 60% confluence (namely primary cells (P0)), the cultures were harvested with trypsin/EDTA 1:1 and cell pellets were obtained by centrifugation at 1,200 rpm to obtain passage one cells (P1). Thereafter, for subsequent passages, each culture was grown to 50–60% confluence (about 10 days), digested by trypsin/EDTA, washed and placed 1:4 into fresh PLL coated 75-cm^2^ flasks as described.

CK19, Sca-1, CD133 and DLK markers were used at each passage to identify the cells as stem cells, and EpCAM, CD14, CD24 and CD49f as markers of liver progenitors^40^,^78^. The cells were seeded onto PLL-coated coverslips, 75-cm^2^ flasks or 6-well plates, and grown for 3 days unless indicated differently. Cell purity was determined by NG2 antibody staining and the cells were passaged at least once before use. These NG2^+^ cells were named MLpvNG2^+^ cells (mouse liver portal perivascular region).

### Liver cirrhosis induction and MLpvNG2^+^ cells transplantation

Liver cirrhosis was induced using diethylnitrosamine (DEN) (Sigma, NO756), as previously reported^35^. Mice received normal drinking water that contained 0.014% DEN, 6 days/per week for 15 weeks. The 1st cohort of 30 mice received DEN for 15 weeks in order to monitor liver progression from normal to fibrotic, cirrhotic, and eventually liver cancer (n = 30), as identified by Masson’ Trichrome, picroSirius Red and hematoxylin-eosin (H&E) stainings. Both Masson’s Trichrome and Picrosiriusis are the protocols used in histology for distinguishing cells from surrounding connective tissue such as collagen fibers. The blue or green color of Masson’s Trichrome stained for collagen fibers and the red color of picroSirius Red stained for reticular fibres, the basal laminae of capillaries, the birefringence of collagen fibers and co-aligned molecules of Type I collagen, which may be obscured by Masson’ Trichrome staining. The 2nd cohort of 37 mice was assigned to receive treatment. Prior to transplantation, MLpvNG2^+^ cells were labeled with carboxyfluorescein diacetate succinimidyl ester (CFSE)^71^. At 6 weeks of DEN administration, mice were randomly divided into the DEN and phosphate buffered saline (PBS) group (DEN^+^ PBS) (n = 13), or the DEN and MLpvNG2^+^ cell group (DEN^+^ MLpvNG2^+^) (n = 13). An additional group of age-matched male mice (n = 11) were used as naïve controls. For the DEN^+^ MLpvNG2^+^ group, mice were given a tail vein injection of CFSE-labeled MLpvNG2^+^ cells (1 × 10^6^ cells in 200 μl for each mouse) at 6 weeks of DEN administration. Animals in the DEN^+^ PBS group received the same volume of PBS (200 μl for each mouse). Both groups continuously received DEN and were monitored for an additional 4 weeks. Upon sacrifice by cervical dislocation, liver samples from each animal were collected and processed: (a) fixed in 4% buffered formaldehyde and embedded in paraffin; (b) snap frozen at −80 °C for sectioning and immunohistochemistry or RNA isolation; and (c) homogenized in appropriate buffer (s) for preparation of cirrhotic conditioned media and for biochemical assays.

### Preparation of liver tissue homogenate and modulation of MLpvNG2^+^ cell differentiation

Following sacrifice, liver tissues were collected and washed thrice in ice-cold PBS at 4 °C (pH 7.4, one liver/2 ml) and homogenized with a tissue homogenizer. After centrifugation at 170 × *g* for 10 min, the supernatant was collected and diluted 1:40 with DMEM/F12 media and then passed through a 0.45 μm filter. The filtered supernatant from livers of the naïve control, DEN^+^ PBS, and DEN^+^ MLpvNG2^+^ groups were named Nai-hm (from naïve control mouse livers), Cir-PBS-hm (from cirrhotic mouse livers treated with PBS) and Cir-cell-hm (from cirrhotic mouse livers treated with cells). Protein concentrations of each sample were assessed using a Lowry protein assay kit (Beyotime, Haimen, China). MLpvNG2^+^ cells (2 × 10^5^) were plated on a coverslips coated with PLL and cultured in 300 μl (35 μg of protein) of homogenate at 37 °C in 5% CO_2_ for different durations prior to immunohistochemistry.

Additional procedures including cell growth (CCK)-8 kit assay^34^, Masson’ Trichrome staining^36^, H&E staining^37^, qRT-PCR^79^, western blot^80^, assessment of trilineage differentiation^81^ and cell colony formation assay^82^ were performed as previously described, and are available as Supplementary Materials. All primers used in this study are listed in Table 1.

### Statistical analysis

All data are presented as mean ± standard error of the mean (SEM) from at least three independent experiments, and evaluated by Student’s *t* test (unpaired, two-tailed) or one-way analysis of variance (ANOVA). *P*-values < 0.05 were considered significantly different.

## Additional Information

**How to cite this article**: Zhang, H. *et al.* Repair of liver mediated by adult mouse liver neuro-glia antigen 2-positive progenitor cell transplantation in a mouse model of cirrhosis. *Sci. Rep.* **6**, 21783; doi: 10.1038/srep21783 (2016).

## Supplementary Material

Supplementary Information

## Figures and Tables

**Table 1:** Primer sequences used for quantitative real-time PCR.

Gene	Sense (5′–3′)	anti-sense (5′–3′)
*Emr1(F4*^+^ */80)*	TCCTGCTGTGTCGTGCTGTTC	GCCGTCTGGTTGTCAGTCTTGTC
*IL-1β*	GCACTACAGGCTCCGAGATGAAC	TTGTCGTTGCTTGGTTCTCCTTGT
*TNF-α*	GGAACTGGCAGAAGAGGCACTC	GCAGGAATGAGAAGAGGCTGAGAC
*AFP*	AGTTTCCAGAACCTGCCGAG	ACCTTGTCGTACTGAGCAGC
*Albumin*	GCAGATGACAGGGCGGAACTTG	CAGCAGCAATGGCAGGCAGAT
*c-Met*	TGCAACGGAGAAGACTCCTA	TGTGTCCACCTCATCATCAG
*Actb*	CAGATGTCGATCAGCAAGCAGGAG	TCAGTAACAGTCCGCCTAGAAGCA
*HNF1a*	CTGTACACCTGGTACGTCCG	TACGCCCCTTCTTAGTTGGC
*HNF4a*	GCATGGATATGGCCGACTACA	ACCTTCAGATGGGGACGTGT
*GATA4*	TGGAAGACACCCCAATCTCG	AGGTAGTGTCCCGTCCCATC
*HNF3β*	CATGGGACCTCACCTGAGTC	TCCTCCCGACACTGTGATCT
*EpCAM*	AACACAAGACGACGTGGACA	GCTCTCCGTTCACTCTCAGG
*Sox9*	GTGCAAGCTGGCAAAGTTGA	TGCTCAGTTCACCGATGTCC
*TAT*	GCTATGCCCCATCTATCGGC	GCAGCCACTCGTCAGAATGA
*HNF1β*	AGCCAGTCGGTTTTACAGCA	TCCTCCCGACACTGTGATCT
*CK19*	CGGACCCTCCCGAGATTACA	TGGAGTTGTCAATGGTGGCA
*G6Pc*	CAGTGGTCGGAGACTGGTTC	TATAGGCACGGAGCTGTTGC

Note: *Emr1(F4*^+^*/80):* EGF-like module receptor 1; *IL-1β:* interleukin-1 beta; *TNF-α:* tumor necrosis factor-alpha; *AFP*: alpha-fetoprotein; *Alb*: Albumin; *c-Met:* hepatocyte growth factor receptor; *Actb:* beta-actin; *HNF1a:* hepatocyte nuclear factor 1-alpha; *HNF4a:* hepatocyte nuclear factor 4-alpha; *GATA4:* GATA binding protein 4; *HNF3β:* hepatocyte nuclear factor 3-beta; *EpCAM:* epithelial cell adhesion molecule; *Sox9:* SRY (sex determining region Y)-box 9/SRY-box containing gene; *TAT:* tyrosine aminotransferase; *HNF1β:* hepatocyte nuclear factor 1-beta; *CK19:*cytokeratin-19; *G6Pc:* glucose-6-phosphatase catalytic.
