# Supplementary material for: Repair of liver mediated by adult mouse liver neuro-glia antigen 2-positive progenitor cell transplantation in a mouse model of cirrhosis
Source: Sci Rep. 2016 Feb 24;6:21783. doi: 10.1038/srep21783 (PMC4764864; doi:10.1038/srep21783)
Supplement: Supplementary Information [file srep21783-s1.doc]

**Repair of liver mediated by adult mouse liver neuro-glia antigen 2-positive progenitor cell transplantation in a mouse model of** **cirrhosis**

Hongyu Zhang^1*^, Siegel Christopher^2*^, Ling Shuai^1*^, Jiejuan Lai^1^, Linli Zeng^1^, Yujun Zhang^1^, Xiangdong Lai^1^, Ping Bie^1§^ , Lianhua Bai^1§^

^1^Hepatobiliary Institute, Southwestern Hospital, No 30. Gaotanyan, ShapingBa Distract, Chongqing 400038, China

^2^Department of Surgery, Division of Hepatobiliary and Abdominal Organ Transplantation, Case Western Reserve University Hospital, Cleveland OH 44106, USA

^*^These authors contributed equally to this study.

**^§^CORRESPONDING AUTHORS:**

**Lianhua Bai**

No. 30 Gaotan Yan, ShapingBa District, Chongqing, 400038 China

Tel: +86-23-68765808

Fax: +0086-2365462170

E-mail: [qqg63@outlook.com](mailto:qqg63@outlook.com)

**Ping Bie**

No. 30 Gaotan Yan, ShapingBa District, Chongqing, 400038 China

Tel: +86-23-68754168

Fax: +0086-2365462170

E-mail: [medscibp@126.com](mailto:medscibp@126.com)

**SUPPLEMENTARY MAIN TEXT**

**Immunohistochemistry and microscopy**

Cell cultures were fixed in 5% acid methanol (-20°C) for 12 min and washed twice for 5 min with room-temperature DMEM with 5% normal goat serum (NGS). Cultures were incubated with antibodies against NG2, platelet-derived growth factor receptor beta (PDGFR-β), CK19, Sca-1, CD133, DLK, CD31, CD34 and CD45 (Abcam, Cambridge, MA) (diluted 1:100 in DMEM) for 30 min at 37°C in a humidified chamber and washed 6 times for 5 min in DMEM with 5% NGS, followed by incubation with fluorescent conjugated secondary antibodies (Sigma, St Louis, MI, 111-545-003) diluted 1:200 in DMEM with 5% NGS for 30 min at 37°C, washed briefly and mounted in Vectashield with DAPI (Sigma, St Louis, MI). The proportion of positive cells for each different cell markers relative to the total number of total NG2- or DAPI-positive cells were counted by an observer blinded to the treatment from six randomly selected fields taken from at least two different coverslips from four separate preparations. The data were pooled and presented at mean ± standard deviation.

For proliferation assays, MLpvNG2^+^ cells were grown until they reached 75% confluence. 5-bromo-2'-deoxyuridine (BrdU) (Roche, Indianapolis, IN, 11170376001) was added 18 h before fixation. Cells were washed with DMEM/F12 and double-labeled for NG2 and BrdU. Each experiment was repeated at least 3 times with data from duplicate coverslips. Proliferation was assessed by quantifying the number of BrdU positive cells as a proportion of the total number of NG2^+^ cells.

Animals were perfused with 4% paraformaldehyde and selected tissues cryoprotected in 30% sucrose overnight, frozen in OCT mounting medium, and sectioned (20 μm) on a Leica cryostat. Primary antibodies against HSC (α-SMA), Kuppfer cells (F4^+^/80), α-fetal protein (AFP) and albumin (Alb) were bused in addition to stain with hematoxylin and eosin (H&E) (Beyotime, c0107) for infiltration cells ^1^. All stained cells and sections were observed with a Leica microscope (DM5500B, Leica, Germany) and analyzed with Leica Microsystem (CH-9435)

**FACS analysis**

Isolated MLpvNG2^+^ cells were incubated with FITC-conjugated antibodies against NG2 (BD Pharmingen, San Diego, CA), EpCAM, CD14, CD24, CD49f and vWf for 30 min at 4ºC. Cells were analyzed on a FACSCaliber (BD Biosciences, San Jose, CA) flow cytometer. The data were processed using BD FACSComp™ software.

**Cell count kit (CCK)-8 assay ^34^**

CCK-8 (Beyotime institute of biotechnology, Japan) was used to determine in vitro growth of MLpvNG2^+^ cells. The cells were seeded in 96-well plates (2000 cells/well) and incubated in a humidified atmosphere containing 5% CO_2_ at 37ºC. After 24 h of incubation, cells were washed twice with PBS and incubated in 100 mL of DMEM/F12 plus 5% FBS (GIBCO, Invitrogen Inc., Carlsbad, CA) containing 10 mL CCK-8 reagent for another 3 h. The absorbance of each well was measured at 450 nm using a micro-plate ELISA reader (Varioskan Flashl, Thermo, USA). Culture medium (DMEM/F12 plus 5% FBS) without cells was used as blank control ^79^.

**Quantification of fibrosis**

Paraffin liver tissue sections were prepared at 5 μm in thickness. Areas of fibrosis in each slide were stained with the Masson’ Trichrome staining kit according to the procedure provided by the manufacturer (Biotech Pharmaceutical Co. Ltd, China, KGMST-8003) ^36^. Assessment of fibrotic areas was conducted using a BIOREVO BZ-9000 and BZ-H1C analyzer equipped with the Image J software (Keyence, Japan) at a magnification of 100X. This analyzer measures the mean value of 10 randomly selected areas per sample and calculates the ratio of connective tissue to the whole area of the liver tissue per slide.

**RNA extraction and quantitative real-time polymerase chain reaction (qRT-PCR) analysis** ^79^

Total RNA was prepared from liver tissue using TRIZOL reagent (Invitrogen, Carlsbad, CA), according to the manufacturer’s instructions. The quantification of RNA was determined with a NanoDrop spectrophotometer (Thermo Scientific, Waltham, MA). Reverse transcription and PCR were performed (in triplicate reactions) using the SYBR®-Green PCR Master Mix (Takara Biotechnology, Takara Dalian, Japan) on a Bio-Rad CFX96^TM^ Real-Time PCR Detection System as reported previously. Primers used in these determinations are listed in **Table 1**. Values of target genes were normalized to the expression of the reference gene (β-actin). The results are expressed as fold-change based on the (delta delta Ct, ddCt) calculation.

**Western blot** ^80^

Western blot was performed as described previously. Liver sections were lysed in RIPA buffer [50 mM Tris-HCl, 150 mM NaCl, 1.0% Triton X-100, 0.5% sodium deoxycholate, 1.0 mM sodium orthovanadate, 0.1% sodium dodecyl sulfate (SDS), 1.0 mM phenylmethyl-sulfonylfluoride, 1.0 mg/ml aprotinin, 10 mg/ml leupeptin, and 1.0 mg/ml pepstatin A, pH 7.4] for 30 min on ice. Tissue lysate was centrifuged at 13,000 g for 15 min at 4°C. The supernatant was collected for determination of protein concentration using the Lowry protein assay kit. Prepared tissue lysate samples (30 mg protein each) were electrophoresed on sodium dodecyl sulfate-polyacrylamide (SDS/PAGE) gels and transferred onto nitrocellulose membranes. Membranes were blocked overnight with 5% milk (Wuhan Boster Biological Techology, Cat no ARO104, China) in 0.1% TBST (Tris-Buffered Saline and Tween20) and then sequentially incubated with specific primary antibodies against Alb, TNF-α, (Proteintech, Chicago, IL), F4^+^/80, IL-1β, (Sigma, St Louis MO) and the horseradish peroxidase (HRP)-conjugated secondary antibody. Immunoreactive bands were visualized with an enhanced chemiluminescence (ECL) detection kit (ECL Millipore, Billerica, MA). The absorbance of positive bands was analyzed with the Image J Software.

**Assessment of liver function**

Serum was isolated from the blood samples obtained from each animal. Changes in alanine aminotransferase (ALT), aspartate aminotransferase (AST), Alb, and Total Bilirubin (TLB) samples in serum were measured using a Beckman-Coulter Uni Cel/DxC800 Synchrony Clinical Systems (South Kraemer Blvd, Brea, United States). Changes in Ureagenesis (URE), Cytochrome P450 (CytP450) and Low-density Lipoprotein (LDL) samples in liver tissue homogenate were determined with enzyme-linked immunosorbent assay (ELISA) kits (Nanjing Jiancheng Biological Technology, Inc., China) according to the manufacturer’s instructions. ELISA was performed in triplicate for each sample.

**Assessment of trilineage (adipogenesis, osteogenesis, chondrogenesis) differentiation of MLpcNG2^+^ cells** ^81^

MLpcNG2^+^ cells were cultured in DMEM at the density of 25,000 cells/ml/well in 6-well culture plate. When cells reached confluence, the medium was replaced with either the adipogenic differentiation medium (AM, DMEM containing 10% of FCS, 0.5 μg/ml of insulin, 10 nM of dexamethasone, and 0.5 mM of isobutylmethylxanthine) or the osteogenic differentiation medium (OM, DMEM containing 10% of FCS, 0.3 mM of ascorbic acid, 10 mM of β-glycerophosphate, and 10 nM of dexamethasone) or the chondrogenic differentiation medium (high glucose (4.5 g/l) DMEM with 6.25 g/ml insulin, 6.25 g/ml transferrin, 6.25 g/ml selenous acid, 5.33 g/ml linoleic acid, 1.25 mg/ml bovine serum albumin, 0.1 M dexamethasone, 10 ng/ml TGF-1, 50 g/ml ascorbate 2-phosphate, 2 mM pyruvate and P/S). Cells were treated with either vehicle (0.1% of dimethylsulfoxide) or different concentrations of Icaritin (0.1-10μM) during the process of differentiation. Cell activities for adipogenesis and osteogenesis were monitored with Oil Red O staining and detection of calcium deposition, respectively.

**Colony formation unit (CFU) assay** ^82^

The stemness of the isolated MLpvNg2+ cells was assessed using a colony-forming assay. A total of 500 MLpvNG2^+^ cells per well were seeded into a PLL-coated 6-well plate (Falcon, BD Biosciences, Franklin Lake, NJ, USA) and cultured in complete medium. Colonies were counted using a microscope (SMZ25/SMZ18, Japan).

**SUPPLENMENTARY FIGURES**

**Supplenmentary Fig. 1**  **Colony-forming-unit （CFU）assay.** Number of MLpvNG2^+^ cell CFU was recorded and photographed at day 0, day 1, day 5 and day 10 (n = 4). Scar bar = 50 μm.


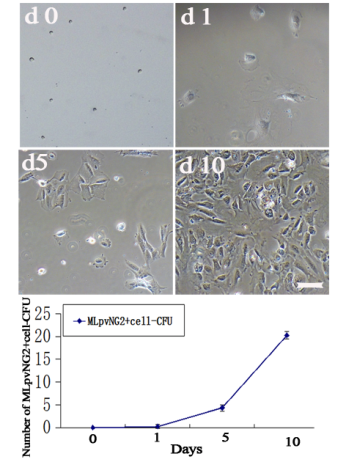


**Supplenmentary Fig. 2 Bile duct cell differentiation of MLpvNG2^+^ cells in response to cirrhotic cues. (a)** MLpvNG2^+^ cells labeled by anti-NG2 antibody (green). (b) bile duct cells stained by anti-CK19 monoclonal antibody. (c) Merged image of (a) and (b). (d) bright-field of the double staining of NG2 with CK19 (n = 3). Scale bar =100 μm. Data are shown as mean ± SEM, ***P*<0.05 *vs.* Nai-hm.


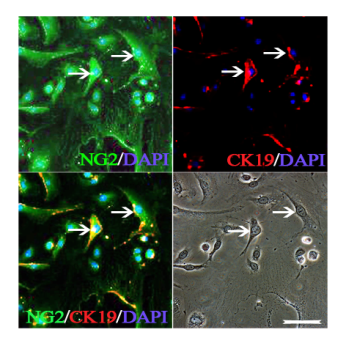


**Supplementary Fig. 3 The anatomic location of periportal vascular region in the liver.** (A) Periportal vascular region, indicated as circles; (B) Any attached parachymal tissues was pealed from the ressected tissue blocks; (C) Two pieces of periportal vascular region tissues were mixed and digested as normal procedure.


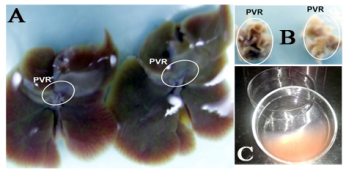


Supplementary Table. Comparison of surface markers and gene expression in native mice

| **Surface markers** | **MLpvNG2^+^**  **/CirNG2^+^cells** | **MSCs**  **/BM-MSCs** | **Oval cells** | **Hepatoblasts** | ------ |
| --- | --- | --- | --- | --- | --- |
| NG2 | +++  [our unpublished work] | +[1] | - | - |  |
| Sox2 SRY | - | +[11] | - | - |  |
| Sca-1/Ly6-A/E | ++ | ++[2] | ++[3] | - |  |
| CD133 | + | - | +[4] | - |  |
| DLK/Pref-1 | + | +[2] | -[10] | +[5] |  |
| EpCAM | + | - | +[22] | +[6] |  |
| CD14/CD11b | + | - | - | - |  |
| CD24 | + | -[12] | +[22] | - |  |
| CD49f  CD49e | +  - | +[13]  +[7] | - | - |  |
| CD45 | - | - [12] | -[4] | -[14] |  |
| CD31 | - | -[8] | - | - |  |
| vWF | - | - | - | - |  |
| CD34 | - | -[24] | +[24] | - |  |
| CD144 | - | -[24] |  |  |  |
| CL19 | - | - |  |  |  |
| Albumin | - | - |  |  |  |
| AFP | - | - |  |  |  |
| A6 | - | - | ++[23] | - |  |
| AFP | - | -  [our unpublished work] | -[10] | - |  |
| PDGFR-β | ++ | +[9] | - | - |  |
| CD146 | ++ | ++[24] | - | - |  |
| CD90 | + | ++[24] |  |  |  |
| CD140b | + | ++[24] |  |  |  |
| PDGFR-α | - | +[15] | - | +[14] |  |
| **Gene expression**  (qRT-PCT) | **MLpvNG2^+^**  **/CirNG2^+^cells**  (our unpublished work) | **MSCs**  **/BM-MSCs**  (our unpublished work) | ------ | ------ | **HybHB**  [16] |
| *NG2/CSPG4)* | + | +[17] | - | - | unknown |
| *Sox2 SRY* | **-** | +[18] | - | - | unknown |
| *Sox9 SRY* | ++ | + | - |  | + |
| *CK19* | + | ++ |  |  | - |
| *EpCAM* | + | ++ |  |  | + |
| *Lgr5* | + | ++ |  |  | + |
| *OPN* | + | - |  |  | + |
| *HNF1β* | + | ++ |  |  | + |
| *HNF1α* | ++ | + |  |  | + |
| *HNF4α* | ++ | - |  |  | + |
| *Alb* | ++ | + |  |  | + |
| *AFP* | + | - | +[16] |  | - |
| *CD56(NCAM)* | - | - | +[16] | - | - |
| *CD117(c-Kit)* | - | +[19] | +[16] | - | - |
| *HGF* | + | +++ | +[3] | +[20] | - |
| *c-Met(MET)* | +++ | + | +[21] | - | - |

**Note:** +: low expression; ++: normal expression; +++: high expression; -: negative or unknwon. Sox2 SRY: sex determining region Y)-box 2; Sox9 SRY: sex determining region Y)-box 9; Sca-1/Ly6-A/E: Stem cell antigen-1; DLK/Pref-1: Protein delta homolog; EpCAM: Epithelial cell adhesion molecule; vWF: Von Willebrand factor; AFP: Alpha fetal protein; PDGFR-β: Platelet-derived growth factor receptor beta; PDGFR-α: Platelet-derived growth factor receptor alpha; Lgr5: Leucine-Rich Repeat Containing G Protein-Coupled Receptor 5; OPN: Osteopontin; HNF1β: Hepatocyte nuclear factor 1beta; HNF1α: Hepatocyte nuclear factor 1alpha; HNF4α: Hepatocyte nuclear factor 4alpha; Alb: Albumin; CD56(NCAM): Neural Cell Adhesion Molecule; HGF: Hepatic growth factor; c-Met (MET): HGF) is the only known ligand of the MET receptor.

**References**

1. Kluth, S. M. *et al.* DLK-1 as a marker to distinguish unrestricted somatic stem cells and mesenchymal stromal cells in cord blood. *Stem Cells Dev* **19**, 1471-1483 (2010).

2. Gang, E. J., Bosnakovski, D., Figueiredo, C. A., Visser, J. W. & Perlingeiro, R. C. SSEA-4 identifies mesenchymal stem cells from bone marrow. *Blood* **109**, 1743-1751 (2007).

3. Petersen, B. E. *et al.* Mouse A6-positive hepatic oval cells also express several hematopoietic stem cell markers. *Hepatology* **37**, 632-640 (2003).

4. Rountree, C. B. *et al.* A CD133-expressing murine liver oval cell population with bilineage potential. *Stem Cells* **25**, 2419-2429 (2007).

5. Sugiyama, D., Kulkeaw, K. & Mizuochi, C. TGF-beta-1 up-regulates extra-cellular matrix production in mouse hepatoblasts. *Mech Dev* **130**, 195-206 (2013).

6. Mitra, M. *et al.* EpCAM is a putative stem marker in retinoblastoma and an effective target for T-cell-mediated immunotherapy. *Mol Vis* **18**, 290-308 (2012).

7. Pelekanos, R. A. *et al.* Comprehensive transcriptome and immunophenotype analysis of renal and cardiac MSC-like populations supports strong congruence with bone marrow MSC despite maintenance of distinct identities. *Stem Cell Res* **8**, 58-73 (2012).

8. Peister, A. *et al.* Adult stem cells from bone marrow (MSCs) isolated from different strains of inbred mice vary in surface epitopes, rates of proliferation, and differentiation potential. *Blood* **103**, 1662-1668 (2004).

9. Popova, A. P. *et al.* Reduced platelet-derived growth factor receptor expression is a primary feature of human bronchopulmonary dysplasia. *Am J Physiol Lung Cell Mol Physiol* **307**, L231-239 (2014).

10. Jelnes, P. *et al.* Remarkable heterogeneity displayed by oval cells in rat and mouse models of stem cell-mediated liver regeneration. *Hepatology* **45**, 1462-1470 (2007).

11. Koike, C. *et al.* Characterization of amniotic stem cells. *Cell Reprogram* **16**, 298-305 (2014).

12. Battula, V. L. *et al.* Epithelial-mesenchymal transition-derived cells exhibit multilineage differentiation potential similar to mesenchymal stem cells. *Stem Cells* **28**, 1435-1445 (2010).

13. Lee, R. H. *et al.* The CD34-like protein PODXL and alpha6-integrin (CD49f) identify early progenitor MSCs with increased clonogenicity and migration to infarcted heart in mice. *Blood* **113**, 816-826 (2009).

14. Ito, K. *et al.* Mesenchymal progenitor cells in mouse foetal liver regulate differentiation and proliferation of hepatoblasts. *Liver Int* **34**, 1378-1390 (2014).

15. Pinho, S. *et al.* PDGFRalpha and CD51 mark human nestin+ sphere-forming mesenchymal stem cells capable of hematopoietic progenitor cell expansion. *J Exp Med* **210**, 1351-1367 (2013).

16. Font-Burgada, J. *et al.* Hybrid Periportal Hepatocytes Regenerate the Injured Liver without Giving Rise to Cancer. *Cell* **162**, 766-779 (2015).

17. McKenzie, K. P., Mayer, D. C. & Aubin, J. E. Osteogenesis and expression of the bone marrow niche in endothelial cell-depleted HipOPs. *J Cell Biochem* **114**, 1066-1073 (2013).

18. Kawasaki, T. *et al.* Transient Exposure to Hypoxic and Anoxic Oxygen Concentrations Promotes Either Osteogenic or Ligamentogenic Characteristics of PDL Cells. *Biores Open Access* **4**, 175-187 (2015).

19. Lamoury, F. M., Croitoru-Lamoury, J. & Brew, B. J. Undifferentiated mouse mesenchymal stem cells spontaneously express neural and stem cell markers Oct-4 and Rex-1. *Cytotherapy* **8**, 228-242 (2006).

20. Sugiyama, Y., Takabe, Y., Yagi, S., Koike, T. & Shiojiri, N. Immunomagnetic exclusion of PECAM-1-positive endothelial cells in fetal mouse liver cell cultures causes impaired growth and gene expression of hepatoblasts and stellate cells. *Biomed Res* **35**, 271-283 (2014).

21. Martinez-Palacian, A. *et al.* EGFR is dispensable for c-Met-mediated proliferation and survival activities in mouse adult liver oval cells. *Cell Signal* **24**, 505-513 (2012).

22. Schievenbusch, S. *et al.* Neighbor of Punc E 11: expression pattern of the new hepatic stem/progenitor cell marker during murine liver development. *Stem Cells Dev* **21**, 2656-2666 (2012).

23. Yin, D. Z. *et al.* Mouse A6-positive hepatic oval cells derived from embryonic stem cells. *J Huazhong Univ Sci Technolog Med Sci* **34**, 1-9 (2014).

24. Dorrell, C. *et al.* Prospective isolation of a bipotential clonogenic liver progenitor cell in adult mice. *Genes Dev* **25**, 1193-1203 (2011).
